# EMMPRIN Down-regulating miR-106a/b Modifies Breast Cancer Stem-like Cell Properties via Interaction with Fibroblasts Through STAT3 and HIF-1α

**DOI:** 10.1038/srep28329

**Published:** 2016-06-21

**Authors:** Yonglei Liu, Jingling Zhang, Xiangjun Sun, Meilin Li

**Affiliations:** 1 Research center, Linyi People’s Hospital, Shandong, China; 2 Zhongshan Hospital, Fudan University, Shanghai, China; 3 Department of Surgery, Linyi People’s Hospital, Shandong, China

## Abstract

Extracellular matrix metalloproteinase inducer (EMMPRIN) is a heavily glycosylated protein and expresses in cancer cells widely, which plays important roles in tumor progression. However, the role of EMMPRIN in breast cancer stem-like cell properties by interaction with fibroblasts is not known. In the present study, we investigated the effects of fibroblasts on breast cancer stem-like cells. We found that fibroblasts activated by co-cultured breast cancer cells produced higher levels of EMMPRIN, which stimulated the stem-like cell specific, self-renewal and sphere-forming phenotype in breast cancer cells. Increased EMMPRIN expression in activated fibroblasts increased the expression of STAT3 and HIF-1α and showed cancer stem-like cell features in breast cancer cells. We also found that EMMPRIN could down-regulate miR-106a and miR-106b expression in breast cancer cells, which led to activating STAT3 and enhancing HIF-1α expression. Our results illustrated that EMMPRIN has an important role in breast cancer stem-like cells by activation STAT3/HIF-1α through interaction with cancer cells and fibroblasts. The study for the first time indicated that cancer cells and fibroblasts interaction promotes breast cancer cells showing stem-like cells through up-regulation EMMPRIN, and led to inhibiting miR-106a/b expression which targets both STAT3 and HIF-1α expression.

Cancer stem cells (CSC) play important roles in tumor initiation, progression and therapeutic response^1^. The properties of CSC including the self-renewal and differentiation are regulated by many genes or signal pathways in cancer^2^,^3^. More studies showed that solid tumor tissues consist of cellular components and non-cellular components which regulate CSC^2^,^3^. In tumor microenvironment, fibroblasts are the most enriched cells in tumor stroma and play important roles in cancer progression including metastasis, proliferation, anti-apoptosis, angiogenesis and chemoresistance by interaction with cancer cells^4^,^5^,^6^. The activated cancer-associated fibroblasts (CAFs) in the cancer niche build a permissive and supportive microenvironment for tumor development.

Extracellular matrix metalloproteinase inducer (EMMPRIN), also known as CD147 (basigin in mice), is a heavily glycosylated type I transmembrane glycoprotein and expressed widely in tumor cells^7^ and its expression in tumor is usually very high on the surface of various tumors^7^,^8^,^9^,^10^,^11^. EMMPRIN induces several malignant properties associated with cancer, including invasiveness, angiogenesis, anchorage-independent growth and chemoresistance. EMMPRIN is linked to tumor metastasis as it is one of the most constantly upregulated components in bone marrow metastatic cells in lung, prostate and breast cancer^12^,^13^. The most important role of EMMPRIN in fibroblasts and cancer cells interation is that it could promote MMP expression and cancer cells become more aggressive^14^,^15^,^16^,^17^,^18^.

Previous studies suggest that EMMPRIN could promote cancer progression by interaction with fibroblasts in tumor stroma^18^. However, it is still unknown whether EMMPRIN could induce breast cancer cell exhibiting stem-like cells and its molecular mechanism. In the present study, we focus on the regulation of CSCs by stromal fibroblasts, an important cellular component of the tumor-hosting niche in breast cancer. The study indicated that EMMPRIN could down-regulate miR-106a/b which targets STAT3-HIF-1α to promote breast cancer cells showing stem-like cells and may play a fundamental role in regulation of CSC.

## Materials and Methods

### Cell lines and culture

The Breast cancer cell lines including MCF-7, MDA-231, SKBR3, SUM102, ZR75B and BT474 were originally purchased from American Type Culture Collection (ATCC, Manassas, VA, USA) and were maintained in Dulbecco’s Modified Eagle’s Medium containing 10% fetal bovine serum, 100 units/mL penicillin, and 100 μg/mL streptomycin. Non-cancerous human mammary epithelial cells MCF10A were originally purchased from ATCC and were maintained in Dulbecco’s modified Eagle’s medium containing 10% fetal bovine serum, 100 ng/ml EGF, 50 ng/ml Insulin, 100 units/mL penicillin, and 100 μg/mL streptomycin. Fibroblasts Hs578Bst were obtained from ATCC and maintained in Hybri-Care Medium (ATCC, Manassas, VA, USA) with 30 ng/ml EGF, 100 units/mL penicillin, and 100 μg/mL streptomycin. Fibroblasts 1068SK were maintained in Dulbecco’s Modified Eagle’s medium containing 10% fetal bovine serum, 2 mmol/L glutamine, 100 units/mL penicillin, and 100 μg/mL streptomycin. All the cell lines were cultured in a humidified atmosphere of 95% air and 5% CO_2_ at 37 °C.

### Co-culturing of breast cancer cells and fibroblasts and conditioned medium preparation

Fibroblasts were co-cultured with breast cancer cells with the ratio at 1:3. Cells were cultured in DMEM/F12 media with 10% FBS supplemented with 10% FBS in a 37 °C humidified incubator with an atmosphere of 5% CO_2_ and 95% air for 24 hours, and then washed for three times with PBS and finally cultured in 3 ml serum free DMEM/F12 media for 2 hours. Conditioned medium was collected and filtered through a 0.22-μm filter (Merck Millipore, Massachusetts, USA) to remove cellular debris.

### Reagents

Antibody directed against EMMPRIN was obtained from Santa Cruz Biotechnology (TX, USA). Antibody against HIF-1α was purchased from BD (BD Pharmingen, CA, USA). Antibodies of anti-CD44, CD24, Y705-phosphorylated STAT3 (STAT3-Y705), and total STAT3 were obtained from Cell Signaling Technology. All other chemicals were purchased from Sigma-Aldrich. Recombinant human EMMPRIN was purchased from R&D (Minneapolis, MN, USA).

### Western blot analysis

Cells were lysed in a lysis buffer containing 50 mmol/L TRIS-HCl, pH 7.4, 150 mmol/L NaCl, 0.5% NP40, 50 mmol/L NaF, 1 mmol/L Na_3_VO_4_, 1 mmol/L phenylmethylsulfonyl fluoride, 25 μg/mL leupeptin, and 25 μg/mL aprotinin and clarified by centrifugation (14,000 g for 30 min at 4 °C). The protein concentration was determined using the Bradford Coomassie blue method (Pierce Chemical Corp.). Whole-cell lysates were separated by sodium dodecyl sulfate (SDS)-PAGE, transferred onto nitrocellulose, and probed with various primary antibodies and horseradish peroxidase–labeled secondary antibodies. The signals were visualized with an enhanced chemiluminescence detection kit (GE Healthcare).

### EMMPRIN shRNA, STAT3 shRNA, HIF-1α shRNA lentivirus vector construction

shRNA lentiviral particle delivery system was used to generate EMMPRIN, STAT3, and HIF-1α-silenced tumor cell lines according to the manufacturer’s instructions (Sigma-Aldrich, Saint Louis, MO, USA). The lentiviral particles were purchased from Sigma (Sigma-Aldrich, Saint Louis, MO, USA). After selection under puromycin (1 μg/ml), the knocking down effect in the drug resistant cells was evaluated by western blot.

### Tumor spheroid assay

Spheroid forming assays were performed as described. In brief, cells were plated in six-well ultralow attachment plates (Corning Inc., Corning, NY) at a density of 1,000 cells/ml in DMEM supplemented with 1% N2 Supplement (Invitrogen), 2% B27 Supplement (Invitrogen), 20 ng/ml human platelet growth factor (Sigma-Aldrich), 100 ng/ml epidermal growth factor (Invitrogen, Carlsbad, CA, USA) and 1% antibiotic-antimycotic (Invitrogen, Carlsbad, CA, USA) at 37 °C in a humidified atmosphere of 95% air and 5% CO2. Spheroid were collected after 7 days and dissociated with Accutase (Innovative Cell Technologies, Inc.). The cells obtained from dissociation were sieved through a 40-μm filter, and counted by coulter counter using trypan blue dye.

### Immunofluorescent staining of cells

Cells were grown on sterile glass coverslips overnight in a 37 °C culture incubator. Prior to immunofluorescent staining, the cells were fixed in pre-chilled −20 °C methanol for 5 min and then incubated with 10% normal serum in phosphate-buffered saline (PBS) at 37 °C for 30 min to block non-specific binding of IgG. The cells were then incubated with the desired primary antibodies in PBS with 1.5% normal serum at 4 °C overnight. After washing the cells twice with PBS, fluorescence-conjugated secondary antibody and 4′,6-diamidino-2-phenylindole (DAPI, Roche, USA) were added onto the coverslips, and the cells were incubated in the dark at room temperature for 1.5 h. Fluorescently stained cells were examined under a fluorescence microscope.

### Lentivirus carring miR-106a/b

Vectors carrying miR-106a and miR-106b were constructed using the BLOCK-iT pol II miR RNAi Expression Vector Kit with EmGFP (Invitrogen, Carlsbad, CA, USA). The primary miR-106a and miR-106b sequences with flanking regions were amplified by PCR and then coloned into pcDNA6.2-GW/EmGFP-miR. Lenvirus for miR-106a and miR-106b were producted using 293 T cells by co-transfection with lipofectamine-2000 (Invitrogen, Carlsbad, CA, USA) according to the protocol according to the instruction. miR-106a and miR-106b expression was examined using real time RT-PCR.

### Transfection and dual luciferase assay

Cells were seeded on plates and the transfection was carried out in Opti-MEM medium using lipofectamine-2000 according to the manufacturer’s protocol (Invitrogen, Carlsbad, CA, USA). The medium was changed after transfection for 5 h, and the cells incubated at 37 °C for the indicated time. For reporter assays, cells were transfected with the pGL3 basic vector or the control plasmid with co-transfecting with siRNAs. 48 h later, luciferase activity was detected using Dual Luciferase Assay System (Promega, WI, USA) with a Sirius luminometer (Berthold Detection System).

### Real time RT-PCR

Total RNA was extracted from the cells with the indicated treatment using Trizol reagent (Invitrogen) according to the manufacturer’s protocol. RNA was qualified and performed for real time RT-PCR analysis. The primers sequences using in this study were provided in the supplementary material (Table 1). The relative mRNA levels were calculated by comparing Ct values of the samples with those of the reference, all data normalized to the internal control GAPDH. miRNA was reverse transcribed into cDNA using the TaqMan MicroRNA Reverse Transcription kit (Invitrogen).

### Statistics

Data were analyzed by SPSS 13.0 software and presented as mean ± SE of at least three independent experiments. Two-tailed Student’s t test was used for comparisons of two independent groups. *p* < 0.05 was considered statistically significant.

## Results

### Soluble EMMPRIN increases in the conditioned medium from interaction of breast cancer cells and fibroblasts

**Figure 1:**
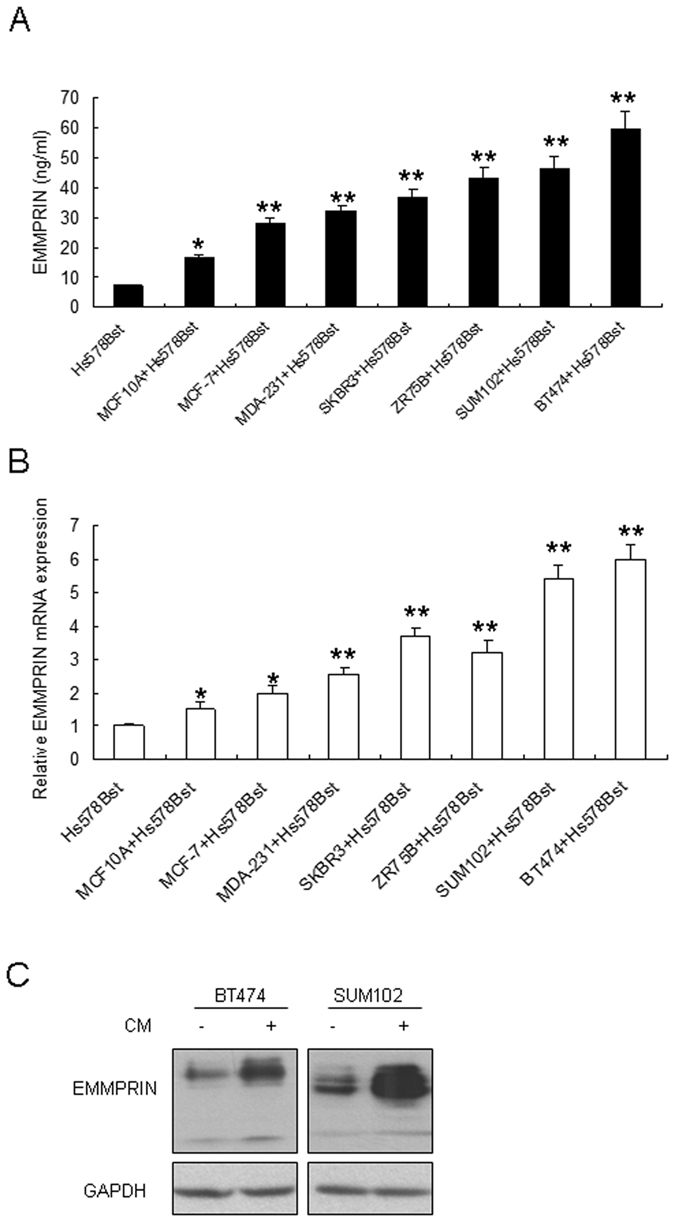
EMMPRIN expression increased in the breast cancer cells with fibroblasts interaction. (**A**) EMMPRIN increased in the conditioned medium (CM) from six breast cancer cells co-cultured with Hs578Bst fibroblasts. EMMPRIN was examined by ELISA. (**B**) EMMPRIN mRNA was enhanced in six breast cancer cell lines with CM from co-cultured cancer cells with fibroblasts compared with CM from fibroblasts. (**C**) EMMPRIN protein levels increased in SUM102 and BT474 cells with CM. CM was collected from the medium of co-cultured breast cancer cells and Hs578Bst fibroblasts. The data presented were shown as means ± s.d. collected from three independent experiments. *p < 0.05, **p < 0.01.

To investigate the role of EMMPRIN in the effect of fibroblasts on breast cancer cells, we examined the soluble EMMPRIN in the conditioned medium from breast cancer cells with fibroblasts interaction. Six breast cancer cells were co-cultured with Hs578Bst fibroblasts and then the conditioned medium (CM) was collected for ELISA analysis. The data showed that soluble EMMPRIN increased in CM from co-cultured breast cancer cells and Hs578Bst cells compared with CM from only Hs578Bst cells or CM from co-cultured MCF10A and Hs578Bst cells (Fig. 1A). At the mRNA level, EMMPRIN mRNA was induced in six breast cancer cell lines significantly in response to CM activation specially in BT474 and SUM102 cells, while in the non-cancerous MCF10A human mammary epithelial cells, there only modestly induced EMMPRIN expression (Fig. 1B). We chosed BT474 and SUM102 to verify the above result, when the cells were treated with CM, total EMMPRIN protein levels in breast cancer cells were enhanced in the two cell lines with CM from co-cultured cancer cells with Hs578Bst fibroblasts (Fig. 1C).

### Recombinant EMMPRIN increases breast cancer cells to show stem-like cell properties

**Figure 2:**
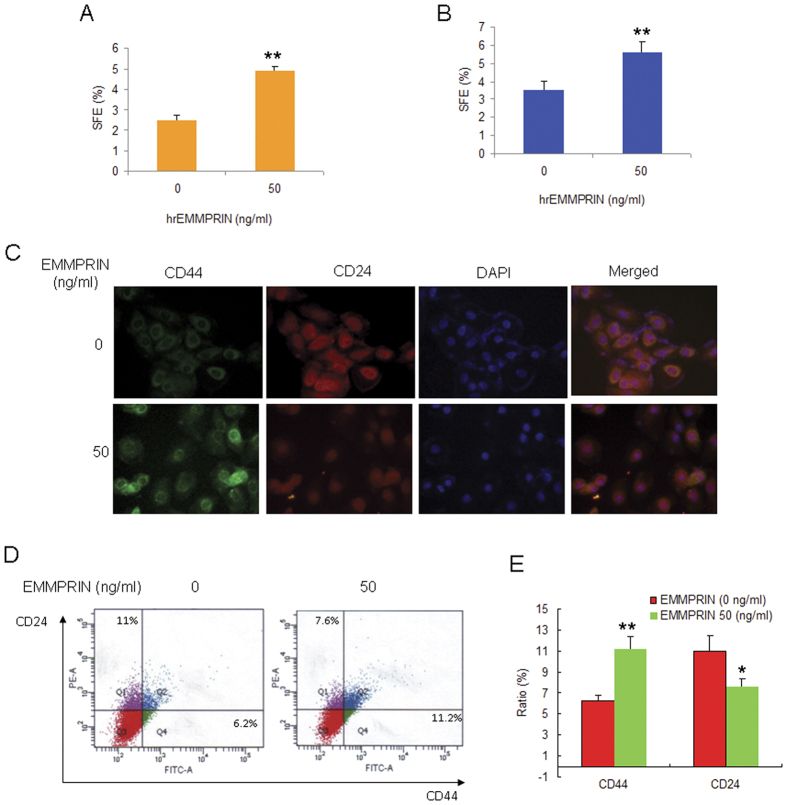
Recombinant EMMPRIN increases breast cancer cells to show stem-like cell properties. EMMPRIN increased the mammosphere formation of breast cancer cells. SUM102 (**A**) and BT474 (**B**) breast cancer cells were treated with hrEMMPRIN and the cells were cultured in stem cell specific medium for 10 days and then numbers of sphere were counted. (**C**) hrEMMPRIN increased CD44 expression and decreased CD24 expression in breast cancer cells. BT474 breast cancer cells were treated with human recombinant EMMPRIN, and then the cells were fixed for immunofluoresence assay. (**D**) hrEMMPRIN increased the percentage of CD44^+^CD24^−^ breast cancer cells. BT474 breast cancer cells were treated with human recombinant EMMPRIN, and then the cells were collected for flow cytometry analysis. (**E**) Data analysis from D. The data presented were shown as means ± s.d. collected from three independent experiments. *p < 0.05, **p < 0.01.

To examine the direct effect of EMMPRIN on breast cancer stem-like cells, SUM102 and BT474 cells were treated with the indicated concentration of human recombinant EMMPRIN (hrEMMPRIN) and stem-like cell properties were analyzed. The mammosphere formation assay indicated that the numbers of mammosphere became more than the control without hrEMMPRIN treatment and showed significant effect at 50 ng/ml hrEMMPRIN (Fig. 2A,B). So, we used 50 ng/ml hrEMMPRIN for the following experiments. To observe whether stem cell markers change in the cells with hrEMMPRIN. The results of immunofluoresence assay indicated that the fluorescence of CD44 enhanced and CD24 reduced in SUM102 cells with hrEMMPRIN treatment comparing with the control (Fig. 2C). The flow cytometry assay also verified the results (Fig. 2D,E).

### Knocking down of EMMPRIN decreases breast cancer cells to show stem like cells properties

**Figure 3:**
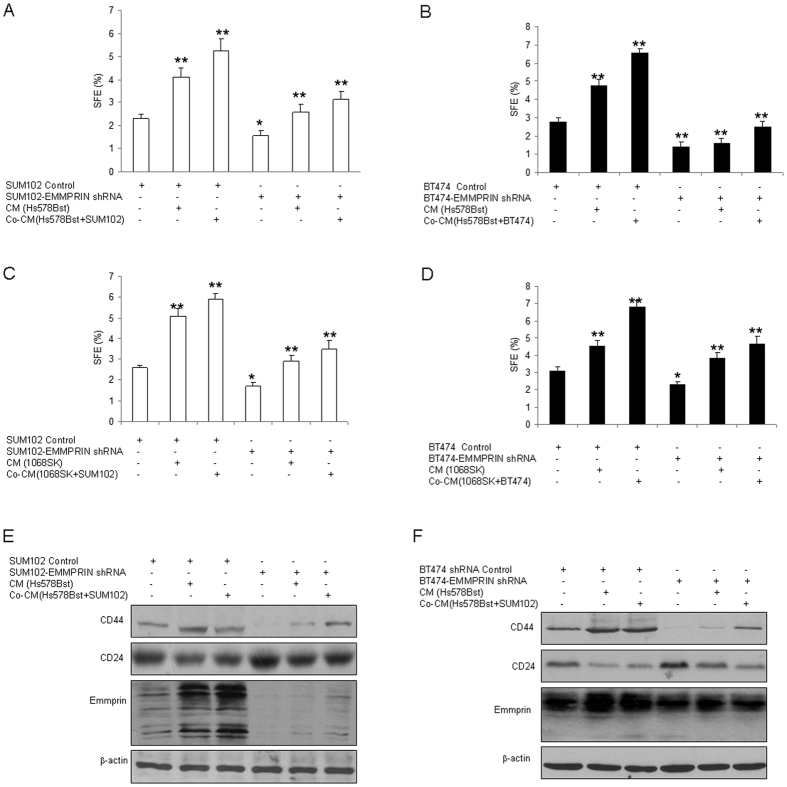
Knocking down of EMMPRIN decreases breast cancer cells to show stem- like cell properties. (**A,B**) EMMPRIN knocking down decreased the percentage of CD44^+^CD24^−^ breast cancer cells. SUM102 and BT474 breast cancer cells were infected lentivirus mediated EMMPRIN knocking down, and the cells were treated with conditioned medium from Hs578Bst fibroblasts for 3 days, and then the cells were collected for mammosphere formation assay. The conditioned medium were collected from Hs578Bst fibroblasts or the breast cancer cells co-cultured with fibroblasts for 3–5 days. (**C,D**) Down-regulation of EMMPRIN decreased mammosphere formation in breast cancer cells. SUM102 and BT474 breast cancer cells with EMMPRIN knocking down or the control were treated with conditioned medium from 1068SK fibroblasts for 3 days and then the cells were digested and used for mammosphere formation assay. EMMPRIN knocking down induced changes of breast cancer stem cell markers. SUM102 (**E**,**F**) BT474 breast cancer cells were infected with LV-EMMPRIN shRNA, and the cells were treated with conditioned medium from fibroblasts 1068SK or Hs578Bst for 3 days, and then the protein were extracted from the cells for western blotting. The data presented were shown as means ± s.d. collected from three independent experiments. *p < 0.05, **p < 0.01.

Above data indicated hrEMMPRIN promoted breast cancer cells to show the properties of breast cancer stem-like cells. Here, we want to know the role of endogenous EMMPRIN in breast cancer cells. SUM102 and BT474 cells were exposed to the conditioned medium from fibroblasts or the co-cultured medium of fibroblasts and cancer. Breast cancer cells with EMMPRIN knocking down were treated with the conditioned medium from the fibroblasts with cancer medium treatment or co-cultured conditioned medium from breast cancer cells and fibroblasts interaction, the results of mammosphere formation assay showed that downregulation of EMMPRIN decreased the sphere formation rates in breast cancer cells by conditioned medium from cancer cell conditioned medium pre-treated Hs578Bst fibroblasts or the co-cultured conditioned medium (Fig. 3A,B). Next, 1068SK fibroblasts were used to verify the result and it was found that there were similar results (Fig. 3C,D). Western blotting showed that stem cell markers CD44 increased and CD24 decreased in the breast cancer cells with conditioned medium treatment, and the ratio of CD44^+^CD24^−^ breast cancer cells became more than the control (Fig. 3E,F). These indicated that EMMPRIN plays a role in promoting breast cancer stem cell formation through interaction of fibroblasts and breast cancer cells.

### STAT3/HIF-1α promotes breast cancer cells to show cancer stem-like cell properties by EMMPRIN

**Figure 4:**
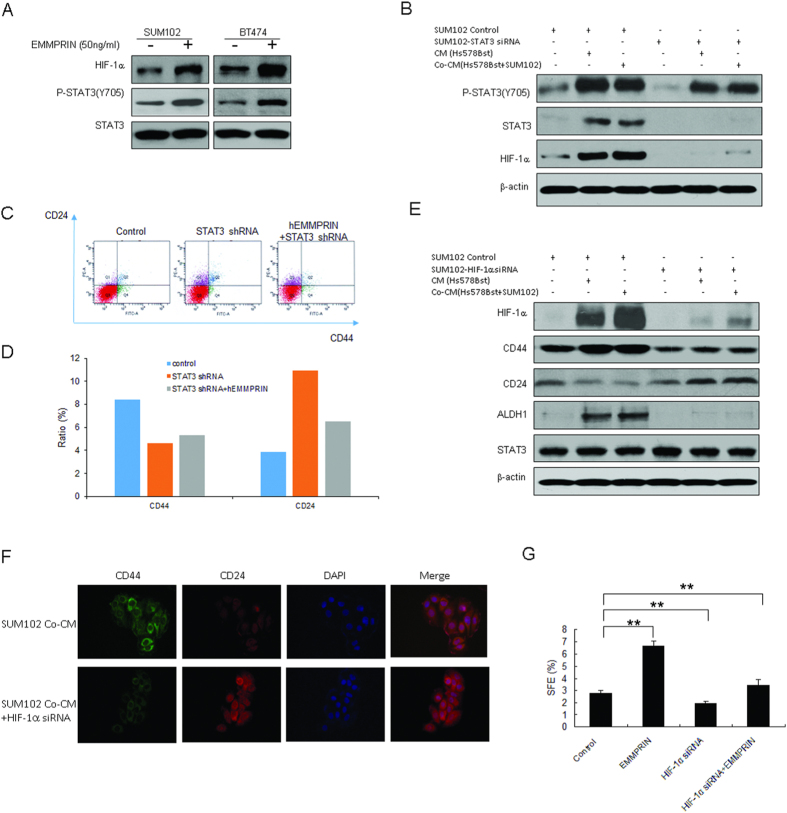
HIF-1α involves in promoting breast cancer stem-like cell properties by EMMPRIN-STAT3 signal pathway. (**A**) SUM102 and BT474 breast cancer cells were exposed to hrEMMRPIN for 24 h and total protein was extracted from the cells for western blotting. (**B**) STAT3 knocking down induced changes of breast cancer stem-like cell markers. STAT3 were knocked down in SUM102 and BT474 breast cancer cells by siRNA, and the cells were treated with conditioned medium from fibroblasts 1068SK or Hs578Bst for 3 days, and then the protein were extracted from the cells. The conditioned medium were collected from fibroblasts or the breast cancer cells co-cultured with fibroblasts for 3–5 days. (**C**) Knocking down of STAT3 decreased the percentage of CD44^+^CD24^−^ breast cancer cells of BT474 treated with human recombinant EMMPRIN by flow cytometry analysis. (**D**) Data analysis from C. (**E**) HIF-1α knocking down induced changes of breast cancer stem cell markers. HIF-1α were knocked down in SUM102 and BT474 breast cancer cells by siRNA. (**F**) HIF-1α knocking down induced changes of breast cancer stem cell markers by immonofluoresence. (**G**) HIF-1α knocking down decreased mammosphere formation in breast cancer cells. The data presented were shown as means ± s.d. collected from three independent experiments. **p < 0.01.

During the investigation of the molecular mechanism underlying EMMPRIN promoting breast cancer stem-like cells, SUM102 and BT474 cells were treated with human recombinant EMMPRIN (hrEMMPRIN) and we found that STAT3 was activated (Fig. 4A). Interestingly, HIF-1α levels increased significantly in the cells with hrEMMPRIN exposure (Fig. 4A). It was also found that STAT3 activity was reduced in SUM102 cells with STAT3 down-regulation, so did HIF-1α protein levels (Fig. 4B). To observe whether hrEMMPRIN has an effect on CD44 and CD24 production, BT474 cells were treated with STAT3 shRNA or hrEMMPRIN. The result showed that CD44 decreased in the cells with STAT3 knockdown, while down-regulation of STAT3 could suppress human recombinant EMMPRIN induced CD44 expression (Fig. 4C,D). So, we speculated that EMMPRIN played a role in stem cells through STAT3. HIF-1α is a subunit of HIF, which plays an important role in cancer progression. HIF-1α can not only be induced by hypoxic stimulation, but also by growth factors and vascular hormones in normoxia. To investigate the enfluence of HIF-1α on the stem cell markers, SUM102 cells were infected LV-HIF-1α shRNA (HIF-1α shRNA) with or without conditioned medium treatment, and the data showed that CD44 level decreased and CD24 level increased (Fig. 4E). The result from immunofluoresence assay indicated that CD44 decreased and CD24 increased in the SUM102 cells with HIF-1α down-regulation (Fig. 4F). When HIF-1α was knocked down in the cells, sphere formation rate was reduced (Fig. 4G). The results showed that HIF-1α could increase the percentage of breast cancer stem like cells via STAT3.

### EMMPRIN attenuates miR-106a/b expression in breast cancer cells

**Figure 5:**
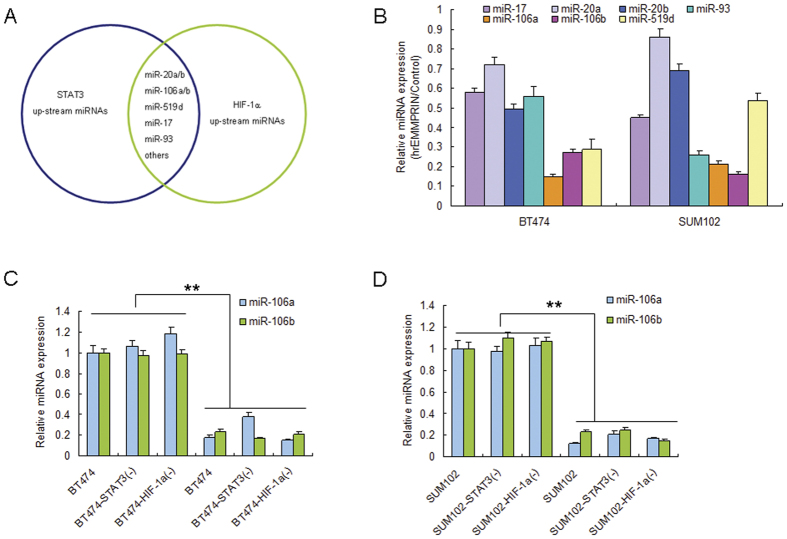
EMMPRIN attentuates miR-106a/b expression in breast cancer cells. (**A**) The predicted target genes of STAT3 and HIF-1α genes contain the binding sites for miRNAs by bioinformatic analysis. (**B**) EMMPRIN decreased miR-106a and miR-106b expression in SUM102 and BT474 breast cancer cells. (**C,D**) breast cancer cells to CM from the co-cultured Hs578Bst and BT474, BT474-STAT3(−), BT474-HIF-1α(−) or SUM102, SUM102-STAT3(−), SUM102-HIF-1α(−) cells. The total RNA was extracted for miRNA expression. The data presented were shown as means ± s.d. collected from three independent experiments. **p < 0.01.

The TargetScan database predicts that both STAT3 and HIF-1α are possibly regulated by miR-106a/b, miR-20a/b, miR-17, miR-93, miR-519d and etc (Fig. 5A). miR-106a/b, miR-20a/b, miR-17, miR-93 and miR-519d were chosen for further reasearch due to their high score and there might have higher possibilities to regulate STAT3 and HIF-1α. BT474 and SUM102 cells were exposed to hrEMMPRIN and the selected miRNAs were analyzed. The results indicated that EMMPRIN could lead to reduction of miR-106a/b expression levels in the cells than miR-20a/b and miR-519d (Fig. 5B). To investigate whether miR-106a/b expression is influenced due to the interaction of fibroblasts and breast cancer cells. Breast cancer cells were exposed to CM from the co-cultured Hs578Bst and BT474, BT474-STAT3(−), BT474-HIF-1α(−) or SUM102, SUM102-STAT3(−), SUM102-HIF-1α(−) cells, and it was found that miR-106a and miR-106b expression were down-regulated in the cells exposure to CM from BT474 or SUM102. However, in the cells with STAT3 or HIF-1α down-regulation, both miR-106a and miR-106b expression were not changed much (Fig. 5C,D).

### miR-106a/b suppresses STAT3 and HIF-1α expression in breast cancer cells

**Figure 6:**
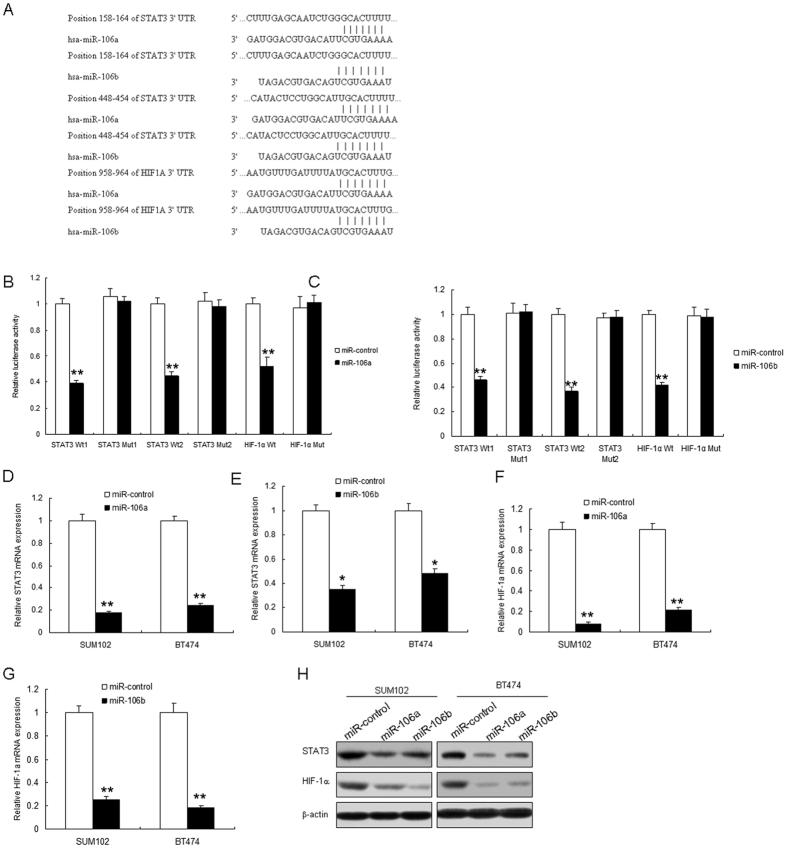
miR-106a/b suppresses both STAT3 and HIF-1α expression in breast cancer cells. (**A**) The 3′-UTR of the STAT3 and HIF-1α genes contains binding sites for miR-106a/b according to bioinformatic analysis. (**B,C**) miR-106a/b suppressed the expression of a luciferase reporter gene harbouring the 3′-UTR of STAT3 or HIF-1α. The pGL3 plasmid was modified by adding the human 3′-UTR or the 3′-UTR with mutations in regions complementary to miR-106a/b seed regions behind the firefly luciferase gene. BT474 cells were transiently co-transfected with miR-control or miR-106a or miR-106b together with the indicated luciferase constructs, and luciferase activity was analysed 48 h later. (**D,E**) miR-106a or miR-106b restoration down-regulated STAT3 mRNA in BT474 and SUM102 cells. Cells were transfected with miR-106a, miR-106b or miR-control for 48 hours, then collected for real-time PCR. (**F,G**) miR-106a or miR-106b restoration down-regulated HIF-1α mRNA in BT474 and SUM102 cells. Cells were transfected with miR-106a, miR-106b or miR-control for 48 hours, then collected for real-time PCR. (**H**) miR-106a or miR-106b restoration down-regulated STAT3 and HIF-1α in breast cancer cells. Cells were transfected with miR-106a or miR-control for 48 hours, then collected for western blot analysis. The data presented are shown as means ± s.d. collected from three independent experiments. *p < 0.05, **p < 0.01.

The bioinformatic analysis showed that 3′-UTRs of the STAT3 and HIF-1α genes contain the binding sites for miR-106a and miR-106b (Fig. 6A). To verify whether STAT3 and HIF-1α are the target genes of miR-106a and miR-106b, plasmids with 3′UTR of STAT3 or HIF-1α were constructed. BT474 cells were transfected with the vectors or miR-106a/b and luciferase activity was assayed. The results showed that the luciferase activity of wide types of pGL3-STAT3 in BT474 cells with miR-106a/b was much lower than miRNA controls (Fig. 6B,C). The luciferase activity of mutated pGL3-STAT3 or pGL3-HIF-1α was rescued in BT474 cells. Endogenous STAT3 and HIF-1α expression in breast cancer cells with miR-106a/b transfection were examined. The results showed that STAT3 decreased when BT474 or SUM102 cells were transfected with miR-106a/b (Fig. 6D,E), so did HIF-1α mRNA (Fig. 6F,G). STAT3 and HIF-1α protein decreased in the cells with the miRNAs (Fig. 6H). Above data showed that STAT3 and HIF-1α were direct target genes of miR-106a/b.

### miR-106a/b attenuates breast cancer stem like cell properties by inhition of STAT3 and HIF-1α

**Figure 7:**
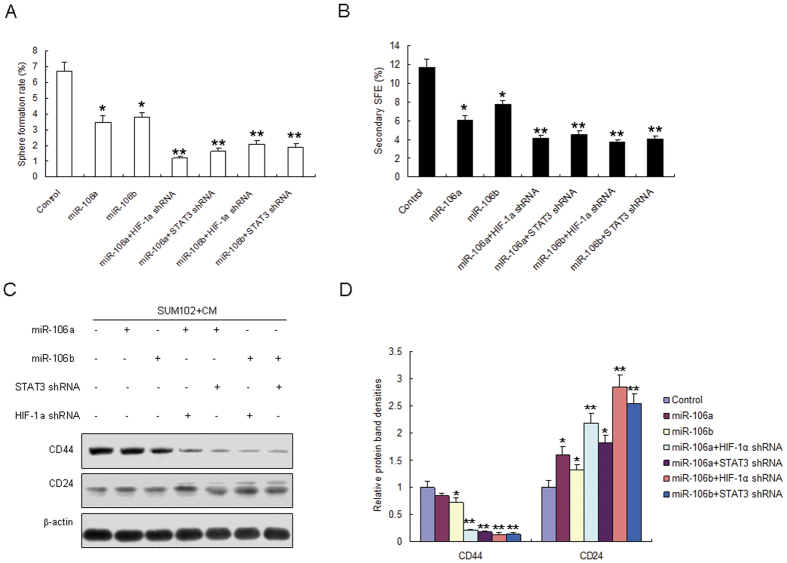
miR-106a/b attenuates breast cancer stem-like cell properties by inhibition of STAT3 and HIF-1α. (**A**) SUM102 cells were tranfected with miR-106a or miR-106b and STAT3 siRNA or HIF-1α in the present of CM from the co-cultured with fibroblasts and cancer cells. Sphere formation was assayed. (**B**) SUM102 cells were tranfected with miR-106a or miR-106b and STAT3 siRNA or HIF-1α in the present of CM from the co-cultured with fibroblasts and cancer cells. The second sphere formation was assayed. (**C**) SUM102 cells were tranfected with miR-106a or miR-106b and STAT3 siRNA or HIF-1α in the present of CM from the co-cultured with fibroblasts and cancer cells. Stem cell markers were examined by western blotting. (**D**) Protein bands from (**C**) were quanlified. The data presented are shown as means ± s.d. collected from three independent experiments. *p < 0.05, **p < 0.01.

To explore the roles of miR-106a/b in breast cancer stem like cell properties by targeting STAT3 and HIF-1α, SUM102 cells were tranfected with miR-106a/b and STAT3 siRNA or HIF-1α in the present of CM from the co-cultured with Hs578Bst fibroblasts and breast cancer cells. It was found that sphere formation rate decreased in the cells with miR-106a/b overexpression (Fig. 7A). The second sphere formation was also decreased (Fig. 7B). The stem markers like CD44 protein levels decreased in the SUM102 cells with miR-106a or miR-106b, but the down-regulation was weaker than in the cells with miRNA-106a/b combinding with STAT3 siRNA or HIF-1α siRNA treatment (Fig. 7C,D). However, CD24 protein levels increased in the SUM102 cells with miR-106a or miR-106b, but the up-regulation was weaker than in the cells with miRNA-106a/b combinding with STAT3 siRNA or HIF-1α siRNA treatment (Fig. 7C,D).

## Discussion

The most riched component fibroblasts could release a set of growth factors, chemokines, and components of the extracellular matrix into the microenvironment and promote cancer progression by altering tumor microenvironment. In the present study, we show that EMMPRIN has a central role in stem-like cell properties of breast cancer through interaction with fibroblasts. Our results illustrate that EMMPRIN has a more fundamental role in breast cancer stem-like cell by down-regulating miR-106a/b and then activation STAT3-HIF-1α through interaction of breast cancer cells and fibroblasts, which for the first time indicated that cancer cell and fibroblast interaction promotes some of brease cancer cells showing stem like cells.

Cancer stem cells (CSC) are a subpopulation of tumor cells that can self-renew, metastasis and promote cancer recurrence. A comprehensive characterization of the CSC proteome has been hampered due to their scarcity and rapid differentiation. Previous study indicated that constitutively expressing high levels of cell-surface EMMPRIN exhibit cancer stem-like cell properties^8^, our data clearly show that in the breast cancer cells with human recombinant EMMPRIN exhibit much greater spheroid formation than the ones without EMMPRIN. When EMMPRIN expression in the breast cancer cells was down-regulated, there were lower sphere formation rates. Futher research showed that breast cancer cells with EMMPRIN knocking-down exhibits a CD44^−^CD24^+^ phenotype and cells with human recombinant EMMPRIN exhibites a CD44^+^CD24^−^ phenotype. Fibroblasts were cocultured with breast cancer cells and transformed to carcinoma-associated fibroblasts by cancer cells. So, we used the conditioned medium from co-culturing cancer cells and fibroblasts pre-treated with cancer cells and they could enhance the ratio of breast cancer stem like cells, and when EMMPRIN expression in breast cancer cells was knocked down, the ratio of breast cancer stem like cells reduced. Breast cancer stem cell properies like self-renew and differenation were shown in breast cancer cells with interaction with fibroblasts.

The STAT3 signaling has been regarded as a critical regulator of tumorigenesis^19^,^20^. Indeed, the strength and duration of STAT3 activation and the formation of feed-forward signaling loops within the tumor stroma are major determinants of cytokine responses and the arising cellular functions that promote tumor growth. Some of the tumor-intrinsic functions of activated STAT3 include: differentiation, cancer stem cell expansion/survival, proliferation, apoptosis and response to hypoxia and cellular metabolism^19^. The STAT3 pathway also regulates tumor-extrinsic aspects of tumorigenesis including: angiogenesis, endothelial cell survival and mesenchymal cell activation and finally progression to metastasis^19^,^20^. Our study indicated that in breast cancer cells with conditioned medium from fibroblasts-breast cancer cells interaction, it could be found STAT3 activation, and down-regulation of EMMPRIN led to reducing STAT3 activation. STAT3 was knocked down in SUM102 and BT474 cells and the stem cell markers CD44 and CD24. One of important findings in our study is that the interaction of fibroblasts-breast cancer cells could stimulate HIF-1α expression. HIF-1α is a known transcription factor in solid tumor stroma, which play critical roles in cancer progression^21^,^22^,^23^.

MiRNAs play important regulatory roles in tumor progression. To explore the role of miRNAs in STAT3-HIF-1α, we used TargetScan to predict the target gene of them. We find that both STAT3 and HIF-1α might be regulated by commen miRNAs such as miR-17^24^, miR-20a^24^, miR-20b^25^, miR-106a^26^, miR-106b^27^, miR-17^24^ and miR-93^28^, which were previously verified as the target genes of STAT3, and miR-20a^29^, miR-20b^30^, miR-17^31^ and miR-93^32^ were previously verified as the target genes of HIF-1α. Interestingly, we found that EMMPRIN suppressed miR-106a and miR-106b expression in breast cancer cells significantly. We also observed that the expression of miR-20a, miR-20b, miR-519d, miR-17 and miR-93, which are the potential reuglator of STAT3 and HIF-1α, but their expression was not influenced by EMMPRIN. The expression levels of the above mentioned miRNAs were inhibited by hrEMMPRIN in SUM102 cells, but their inhibition was not more significant than the expression of miR-106a and miR-106b. This is a new mechanism that EMMPRIN regulates breast cancer stem like cells. Further research showed that miR-106a and miR-106b attenuated sphere formation rates in breast cancer cells exposed to conditioned medium from the co-cultured fibroblasts and breast cancer cells. These suggests that miR-106a and miR-106b may be tumor suppressors in breast cancer progression.

In a conclusion, EMMPRIN plays important roles in breast cancer stem like cells influenced by fibroblasts and cancer cells, inhibits miR-106a/b expression which down-regulates STAT3 and HIF-1α. The study has a significance in guiding breast cancer therapy as targeting EMMPRIN.

## Additional Information

**How to cite this article**: Liu, Y. *et al.* EMMPRIN Down-regulating miR-106a/b Modifies Breast Cancer Stem-like Cell Properties via Interaction with Fibroblasts Through STAT3 and HIF-1a. *Sci. Rep.* **6**, 28329; doi: 10.1038/srep28329 (2016).

## Supplementary Material

Supplementary Information
