# Supplementary material for: EMMPRIN Down-regulating miR-106a/b Modifies Breast Cancer Stem-like Cell Properties via Interaction with Fibroblasts Through STAT3 and HIF-1α
Source: Sci Rep. 2016 Jun 21;6:28329. doi: 10.1038/srep28329 (PMC4914854; doi:10.1038/srep28329)
Supplement: Supplementary Information [file srep28329-s1.doc]

**Supplementary material**

**EMMPRIN Down-regulating miR-106a/b Modifies Breast Cancer Stem-like Cell Properties via Interaction with Fibroblasts Through STAT3 and HIF-1α**

Yonglei Liu^1,2*^, Jingling Zhang^1^, Xiangjun Sun^3^, Meilin Li^2^

1. Research center, Linyi People’s Hospital, Shandong, China

2. Zhongshan Hospital, Fudan University, Shanghai, China

3. Department of Surgery, Linyi People’s Hospital, Shandong, China

Corresponding to: Yonglei Liu, 49 Yizhou Road, Linyi People’s Hospital, Shandong, China. Email: yongleiliu@yeah.net.

Table 1 Primers for qRT-PCR

| Gene | Primer sequence (5’- 3’) |
| --- | --- |
| EMMPRIN | forward primer: TCATGGCGGCTGCGCTGTTCGTG |
| EMMPRIN | reverse primer: TCACGGCCTTCACTCTGGGAG |
| STAT3 | forward primer: ACAGCTTCCCAATGGAGCTG |
|  | reverse primer: ACTCAAGATACCTGCTCTGAAG |
| HIF-1α | forward primer: CCAGATCTCGGCGAAGTAAAGA  reverse primer: CATAACAAAACCATCCAAGGCTTT |
| GAPDH | forward primer:GAAGGTGAAGGTCGGAGTC |
|  | reverse primer: GAAGATGGTGATGGGATTTC |
| miR-17 | forward primer: ACACTCCAGCTGGGCAAAGTGCTTACAGTGC |
| miR-20a | forward primer: ACACTCCAGCTGGGTAAAGTGCTTATAGTGC |
| miR-20b | forward primer: ACACTCCAGCTGGGCAAAGTGCTCATAGTGC |
| miR-93 | forward primer: ACACTCCAGCTGGG CAAAGTGCTGTTCGTGC |
| miR-106a | forward primer: ACACTCCAGCTGGG AAAAGTGCTTACAGTGC |
| miR-106b | forward primer: ACACTCCAGCTGGG TAAAGTGCTGACAGT |
| miR-519d | forward primer: ACACTCCAGCTGGG CCTCCAAAGGGAAGCGCTT |
| URP | reverse primer: TGGTGTCGTGGAGTCG |
| U6 | forward primer: CTCGCTTCGGCAGCACA  feverse primer: AACGCTTCACGAATTTGCGT |
